# Something Old and Something New—A Pilot Study of Shrinkage and Modern Imaging Devices

**DOI:** 10.3390/life15010030

**Published:** 2024-12-30

**Authors:** Josephine V. W. Hearing, Raymund E. Horch, Rafael Schmid, Carol I. Geppert, Maximilian C. Stumpfe

**Affiliations:** 1 Department of Plastic and Hand Surgery and Laboratory for Tissue Engineering and Regenerative Medicine, University Hospital Erlangen, Friedrich-Alexander University Erlangen-Nürnberg (FAU), 91054 Erlangen, Germany; 2 Institute of Pathology, University Hospital Erlangen, Friedrich-Alexander University Erlangen-Nürnberg (FAU), Krankenhausstr. 8–10, 91054 Erlangen, Germany; 3 Comprehensive Cancer Center Erlangen-EMN (CCC ER-EMN), University Hospital Erlangen, Friedrich-Alexander University Erlangen-Nürnberg (FAU), Östliche Stadtmauerstr. 30, 91054 Erlangen, Germany

**Keywords:** shrinkage, tissue oxygenation, temperature, collagen denaturation, wound reduction

## Abstract

Shrinkage, a heat-induced process, reorganizes collagen fibers, thereby reducing wound surface area. This technique, commonly applied in surgeries like periareolar mastopexy and skin grafting, is well-established. Despite its widespread use, modern imaging has recently enabled detailed observation of shrinkage’s effects on tissue temperature and oxygenation. The aim of this study is to investigate the effects of shrinkage on histological level, temperature, and tissue oxygenation. Skin flaps were collected, marked, and subjected to shrinkage in vitro, with wound dimensions recorded before and after shrinkage. Biopsy samples were analyzed histologically. In our clinical set up, Snapshot NIR^®^ and FLIR thermography were used to assess tissue oxygenation and temperature changes before and after shrinkage. Shrinkage significantly reduced wound area by almost 47% ± 8.5%, with a 16.5% ± 6.0% reduction in length and a 36.5% ± 7.7% reduction in width. Tissue temperature rose by an average of 38.3 °C post-shrinkage, reaching approximately 65 °C. A slight decrease in oxygen saturation was observed following shrinkage. Histological analyses reveal collagen fiber denaturation and structural reorganization. Thermal shrinkage is an effective method for reducing wound size and tension, demonstrating potential for facilitating larger full-thickness skin grafts. Although minor decreases in oxygenation were observed, shrinkage may enhance wound healing by reducing tension at wound edges. Further studies are needed to quantify its impact on functional and cosmetic outcomes.

## 1. Introduction

**Figure 1:**
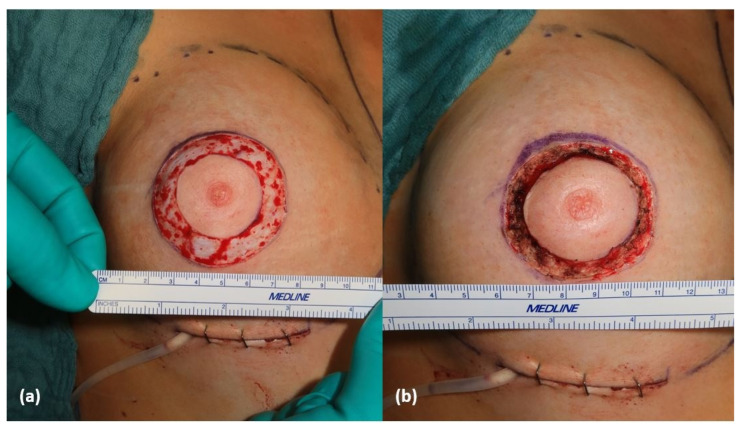
Demonstration of shrinkage at a periareolar mastopexy; (**a**) before and (**b**) after shrinkage, which illustrates the significant reduction in the wound area after the procedure.

Skin is sensitive to heat. Skin’s microstructural organization changes by the application of heat—so called shrinkage [1]. Shrinkage leads to a reorganization of collagen fibers (denaturation) and evaporation (loss of water) [2]. In surgical procedures such as periareolar mastopexy (Figure 1) or full-thickness skin grafting, shrinkage offers a way of reducing the wound surface. The denaturation of collagen is also used in skin rejuvenation for skin tightening [3].

**Figure 2:**
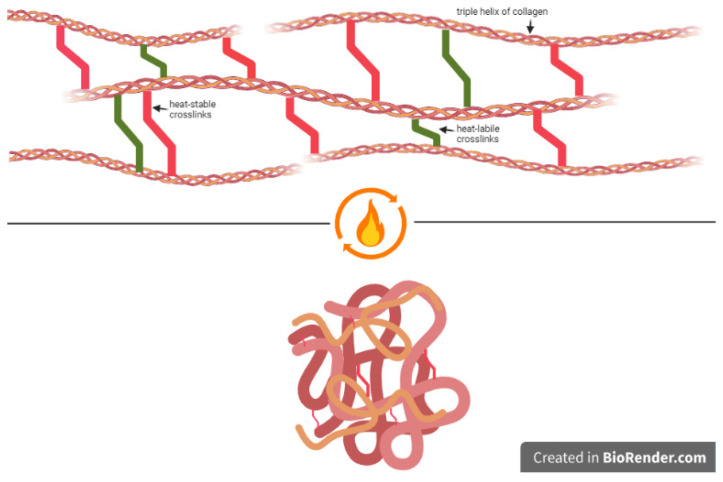
Schematic illustration of shrinkage; heat-labile crosslinks (shown in green) break down at elevated temperatures, while heat-stable crosslinks (in red) remain intact, sustaining the tissue’s structure post-shrinkage. (Modified from Markel, M. D., et al. (2001) [6]—created using BioRender.com, accessed on 5 June 2024).

In general, shrinkage has been used for more than 70 years. Flory et al. proposed and studied the thermal properties of collagen in several studies [4,5,6]. Allain et al. proposed that shrinkage first leads to unwinding of the triple helix, followed by hydrolysis of the heat-labile crosslinks when the temperature rises. The heat-stable crosslinks remain and are responsible for the residual tension (Figure 2) [7,8].

With this study, we combined the well-established technique of tissue shrinkage, often observed in surgery, with advanced imaging modalities for oxygen saturation and tissue temperature. By integrating the histological analysis, we aimed to achieve a comprehensive understanding of the structural and cellular changes induced by shrinkage in skin tissue. This approach allowed us to correlate the effects of shrinkage on wound dimensions with detailed insights into underlying histopathological changes. Our investigation not only contributes to the existing body of knowledge regarding the impact of shrinkage on human tissue but also explores the potential implications for clinical applications and treatment strategies in plastic and reconstructive surgery.

## 2. Materials and Methods

### 2.1. Patients

This study involved patients who underwent medically necessary operations to remove excess skin, with their informed consent. Patients showing signs of skin inflammation were excluded. The study received approval from the Friedrich-Alexander University Erlangen-Nürnberg’s institutional research ethics committee (proposal number 23259-B) and adhered to the 1964 Helsinki Declaration and its subsequent amendments or comparable ethical standards. A preliminary study confirmed no significant differences between in vivo and in vitro results, leading to the experiments being conducted in vitro to minimize operating time for the patients.

### 2.2. Data Acquisition

**Figure 3:**
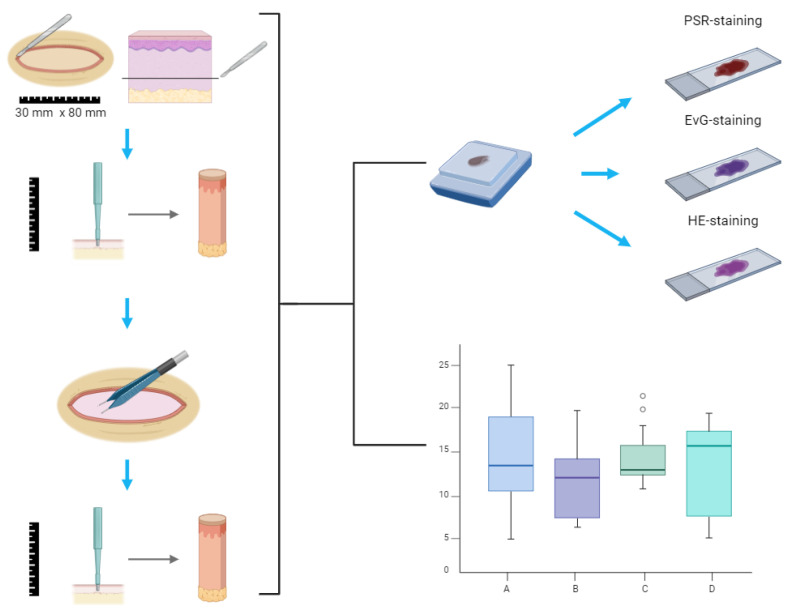
Workflow for samples; tissue sections were prepared and stained with PSR for collagen, EvG for elastic fibers, and HE for overall structure. Wound dimension was statistical compared pre-shrinkage and post-shrinkage; HE-staining = hematoxylin and eosin staining; EvG-staining = Elastica van Gieson’s staining; PSR-staining = picrosirius-red staining (created using BioRender.com, accessed on 5 June 2024).

After surgical removal, skin flaps were immediately transported to the laboratory. The flaps were stretched and secured onto plastic plates until an adequate tension, comparable to the clinical application, was achieved. Spindle shapes measuring 30 mm × 80 mm were marked on the flaps based on their dimensions. A total of 50 pre- and post-shrinkage samples, derived from 50 spindles across 5 patients, were analyzed. The wound sites were measured for length and width before and after shrinkage, shrinkage was performed through coagulation with bipolar diathermy at 5 mV (VIO 3, Erbe Elektromedizin, Tübingen, Germany) evenly over the entire area for about 10 s. A 10 mm punch biopsy was taken from the wound site both pre- and post-shrinkage (Figure 3). Length and width measurements of each spindle were used to calculate approximate wound surfaces pre- and post-shrinkage using the formula for the area of a rhomb (A = ½ × length × width), to evaluate the reduction in size.

### 2.3. Preparation and Digitalization of Histological Sections

Biopsy samples were fixed in 4% formalin (Histofix, Carl Roth, Karlsruhe, Germany), dehydrated and embedded in paraffin, and sectioned at 3 µm. Sections were stained with hematoxylin and eosin (HE), Elastica van Gieson stain (EvG) to highlight elastic fibers, and picrosirius-red stain (PSR) for collagen content analysis according to standard protocols. Hematoxylin and eosin (HE) and picrosirius-red staining (PSR) were performed according to standard protocol by the Institute of Pathology using the Benchmark Ultra (Roche, Basel, Switzerland).

### 2.4. EvG-Staining Was Performed as Follows

The Elastica van Gieson staining procedure begins with deparaffinization by immersing the slides in two changes of xylol for 10 min each. Next, the slides are rehydrated by placing them in two changes of 100% alcohol for 2 min each, followed by two changes of 96% alcohol for 2 min each. After rehydration, the slides are stained with resorcin–fuchsin (Weigert’s method—2E-030, Waldeck, Münster, Germany) for 15 min to highlight elastic fibers, which will appear dark violet. Following the staining, the slides are rinsed in distilled water for 1 min.

For nuclear staining, the slides are treated with Weigert’s hematoxylin (mixed in equal parts A and B—1.15973.0002, Merck KgaA, Darmstadt, Germany) for 3–5 min, with an optimal duration of 4 min. This stains the cell nuclei brown–black. After the staining, the slides are rinsed in distilled water for 2 min to “blue” the tissue.

The next step involves staining with picro-fuchsin van Gieson (2E-050, Waldeck, Münster, Germany) for 5 min. This reagent stains collagen fibers red, muscle tissue yellow, and keratinized epithelium yellow–red. The slides are briefly rinsed in distilled water and acetic acid for 3 s to remove excess dye. Dehydration follows, with the slides immersed in two changes of 96% alcohol for 10 s each, followed by two changes of 100% alcohol for 5 min each.

To clear the tissue, the slides are placed in two changes of xylene for 3 min each. Finally, the slides are mounted using Entellan or an appropriate mounting medium.

The histological sections were analyzed using a standard light microscope (Olympus IX-83 microscope, Olympus, Tokyo, Japan) with the Olympus cellSens Dimension software (version 1.16, Olympus, Tokyo, Japan).

### 2.5. Modern Imaging

A total of 15 patients who underwent medically necessary operations for defect coverage with full-thickness skin grafting were included.

#### 2.5.1. Snapshot NIR^®^

**Figure 4:**
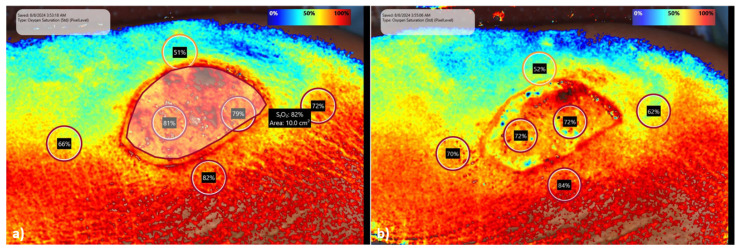
Pre-shrinkage (**a**) and post-shrinkage (**b**) imaging through Snapshot NIR^®^; post-shrinkage images show slight oxygen saturation reductions within the wound area.

Snapshot NIR^®^ (KENT Imaging Inc., Calgary, AB, Canada) is a near-infrared reflectance-based imaging device used to measure tissue oxygenation in superficial tissue. The technique has been described in detail in several studies [9,10]. Briefly, this method transmits light to the skin, where it is selectively absorbed or reflected to calculate the percentage of oxygenated and deoxygenated hemoglobin, thereby assessing tissue oxygen perfusion. Well-perfused skin shows a higher percentage of oxygenated hemoglobin compared to poorly perfused skin, due to the wavelength-dependent differences in light absorption by hemoglobin [9,10]. Snapshot NIR^®^ was performed before and after shrinkage in patients requiring a full-thickness skin graft due to injury. The results were analyzed using three parameters: the exterior border (oxygen saturation values around the external edges of the full-thickness skin donor site), the interior border (oxygen saturation values within the donor site), and the average oxygen saturation across the entire area of the donor site (Figure 4).

#### 2.5.2. FLIR Thermographic Camera

**Figure 5:**
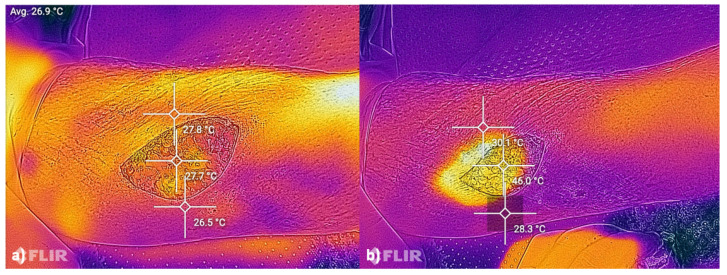
FLIR thermographic images displaying tissue temperatures before (**a**) and after (**b**) shrinkage, indicating the rise in temperature essential for collagen denaturation.

A Cat S61 Smartphone (Caterpillar Inc., Deerfield, IL, USA) with an integrated FLIR-Thermographic camera (FlirOne) was used for thermographic imaging (Figure 5). The temperature ranges were from −20 °C to 400 °C and has a resolution of 0.1 °C [9,10]. FLIR-Thermographic (FlirOne) imaging was similarly accomplished before and after shrinkage.

### 2.6. Statistical Analysis

Descriptive statistics were used to analyze patient demographics. Measurement comparisons were conducted using a paired *t*-test with a two-tailed *p*-value; *p* < 0.05 was considered statistically significant. Data are shown as mean ± SD. The statistics were calculated from 50 technical replicates and 15 patients in routine clinical practice. Statistical analyses were carried out using GraphPad Prism Version 10 software (GraphPad Software, Inc., Boston, MA, USA).

## 3. Results

### 3.1. Patient Demographics

A total of 50 sample pairs (50 spindles, one pre- and one post-shrinkage sample each) originating from five different female patients were analyzed. Patient ages were distributed from 37 years to 66 years (mean average 50 years).

### 3.2. Clinical Wound Sizes

The 50 evaluated spindles had a mean length of 82 mm ± 4 mm (range 75–90 mm) pre-shrinkage and 69 mm ± 6 mm (range 52–78 mm) post-shrinkage, resulting in a reduction of 16.5% ± 6.0%. The mean width was 35 mm ± 2 mm (range 28–40 mm) pre-shrinkage and 22 mm ± 3 mm (range 17–30 mm) post-shrinkage. A 36.5% ± 7.7% reduction has resulted as a consequence of shrinkage. The statistical analysis showed no difference between intra- and interpatient.

**Figure 6:**
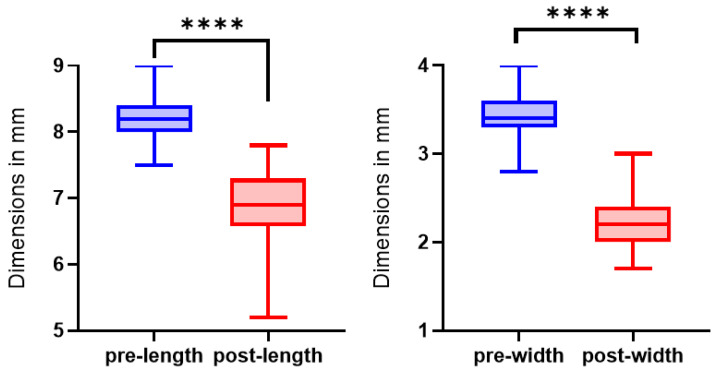
Length and width pre-shrinkage (blue) and post-shrinkage (red) from 50 technical replicas; **** = *p* ≤ 0.0001.

The shrinkage values in both length and width were statistically significant with a *p*-value < 0.0001 (see Figure 6). 

**Figure 7:**
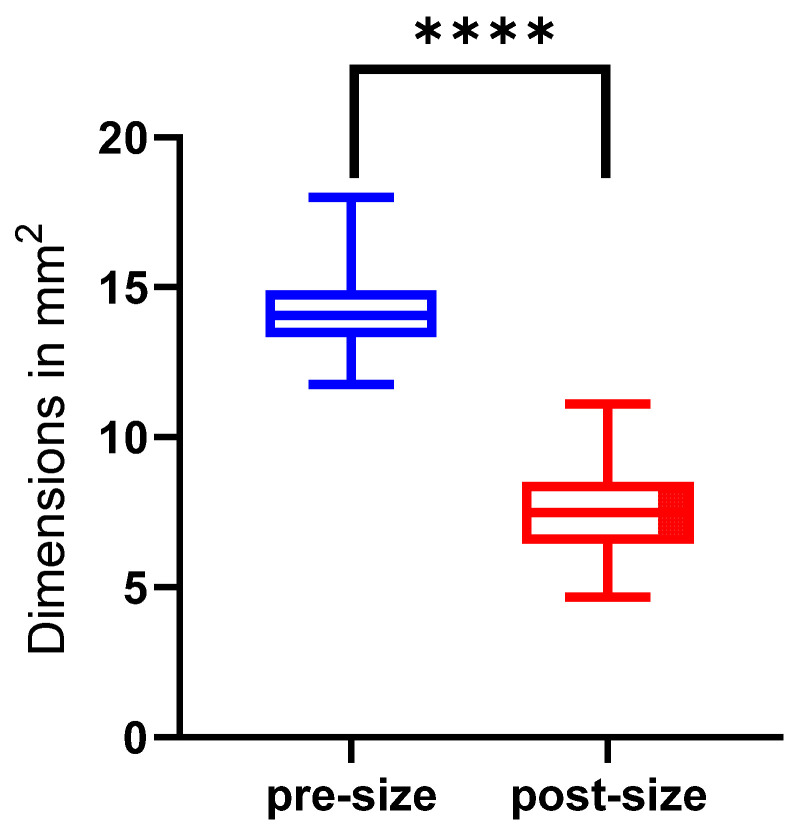
Total wound size pre-shrinkage (blue) and post-shrinkage (red) from 50 technical replicas. **** = *p* ≤ 0.0001.

The mean approximated wound size pre-shrinkage was 1417 mm^2^ ± 115 mm^2^ (range 1176–1800 mm^2^). Post-shrinkage, the mean approximated wound area was 755 mm^2^ ± 138 mm^2^ (range 468–111 mm^2^). Shrinkage achieved an average wound reduction of 46.8% ± 8.5%, which means a significant reduction in size (*p* < 0.0001) (Figure 7).

### 3.3. Histological Analysis

**Figure 8:**
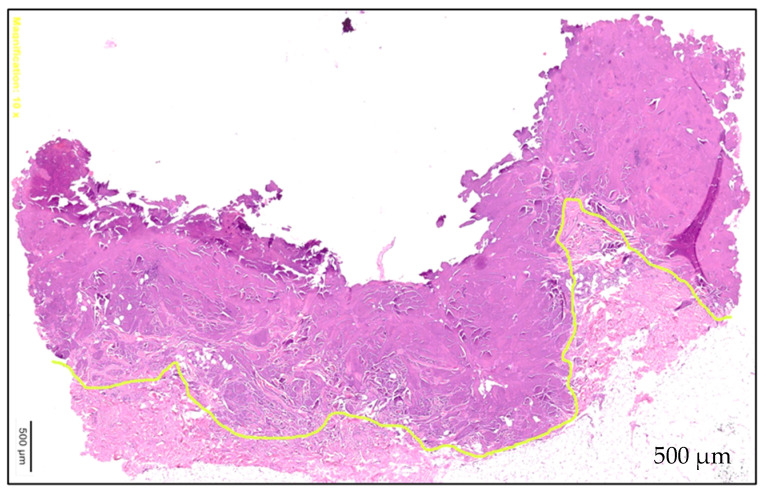
Direct histological comparison between post-shrinkage (upside) and pre-shrinkage (lower part) in HE-staining.

The boundary between tissue affected by shrinkage and unaffected tissue is clearly delineated (Figure 8). There is an overall size reduction of 25% in all samples. 

**Figure 9:**
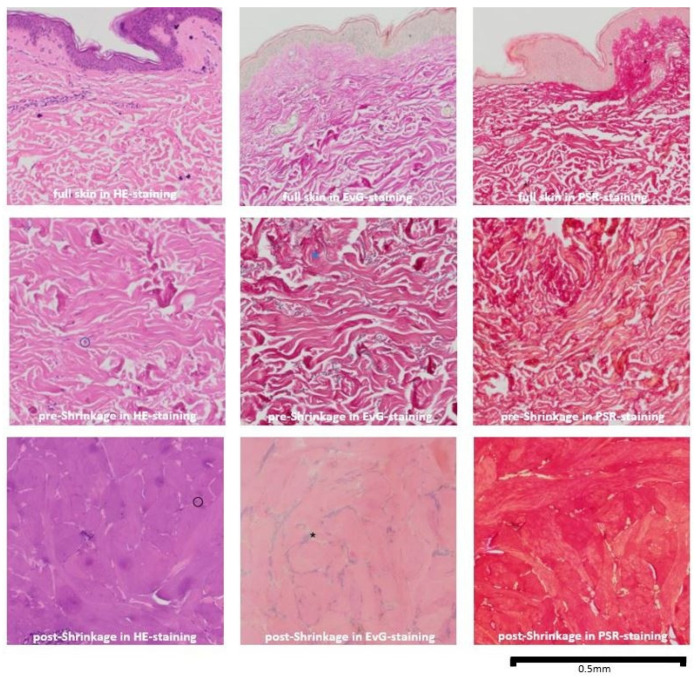
Overview of the individual stains and the comparison between the pre- and post-shrinkage sections; black circle = fragmented nuclei; blue circle = intact fibrocytes with blue to purple nuclei; blue star = intact elastic fibers; black star = coiled elastic fibers; Olympus IX-83 microscope (Olympus, Tokyo, Japan), Magnification 10×.

In the HE-stained sections after shrinkage, the fibrocytes are destroyed, leaving only fragmented nuclei scattered between denatured collagen fibers. These fibers have lost their characteristic wavy appearance and exhibit a disordered arrangement (in contrast to the sections before shrinkage, in which intact fibrocytes with blue–purple cores and red to pink collagen fibers with a wavy, parallel morphology were observed). In the EvG staining sections, the elastic fibers lose their reticular arrangement, appearing coiled between the collagen fibers and are barely visible (compared to the pre-shrinkage sections, where the elastic fibers were clearly visible as thin purple–black filaments in a reticular pattern). In the PSR-stained sections under polarized light, the post-shrinkage sections exhibit a more heterogeneous structure, with thick strands stained dark red and a loss of collagen birefringence (whereas the collagen bundles were alternately stained red and yellow before shrinkage). Figure 9 provides an overview of the individual stains and a comparison between the pre- and post-shrinkage sections.

### 3.4. Modern Imaging Devices

#### 3.4.1. Oxygen Saturation

**Figure 10:**
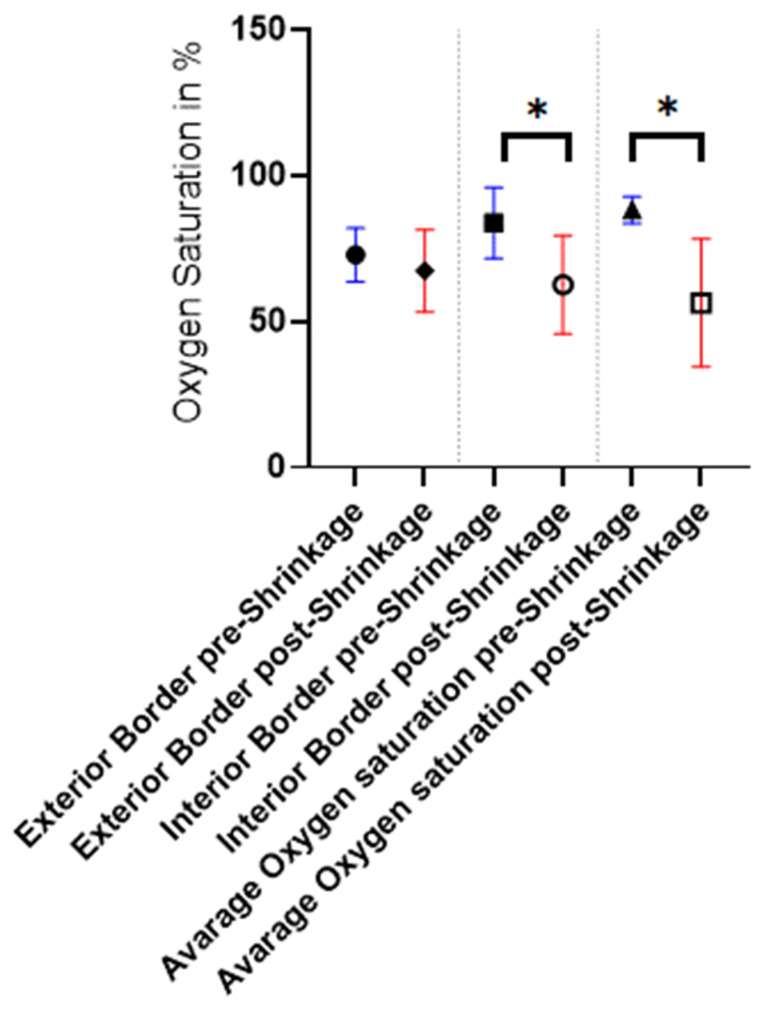
Oxygen saturation pre-shrinkage (blue) and post-shrinkage (red) for the exterior and interior border as well as the entire area after full-thickness skin grafting; y = %; * = *p* ≤ 0.05.

Oxygen saturation was significantly lower after shrinkage compared to pre-shrinkage (see Figure 10). The mean saturation around the donor site was 72.8% pre-shrinkage vs. 67.3% post-shrinkage. The interior border of the defect showed a mean oxygen saturation of 83.7% pre-shrinkage and 62.8% post-shrinkage. The average oxygen saturation of the whole surface dropped from 88.2% to 56.3%. The *p*-value for the interior border is 0.0245 (*) and for the average oxygen saturation 0.0148 (*).

#### 3.4.2. Temperature

**Figure 11:**
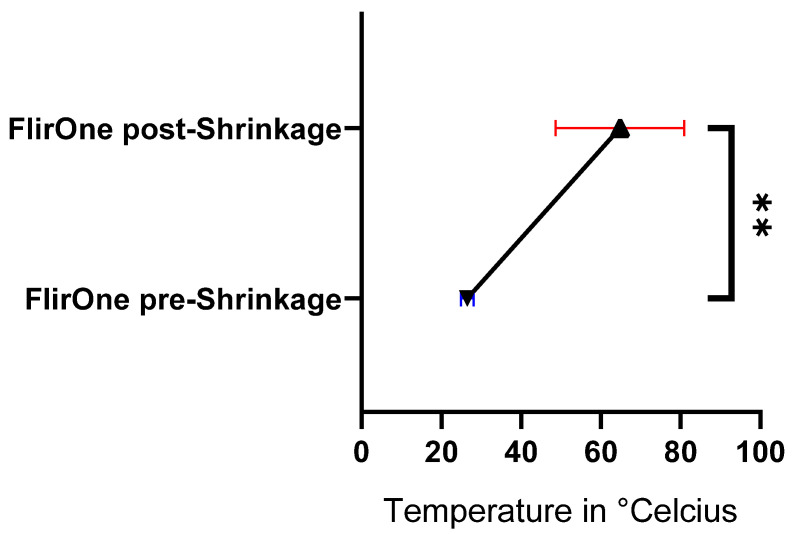
Temperature difference pre-shrinkage and post-shrinkage; ** = *p* ≤ 0.01.

The measured temperature increased after shrinkage. The average maximum temperature was 64.8 °C (range 46.0 °C–87.0 °C). The pre-shrinkage temperature was 26.5 °C. The average rise was 38.3 °C. The post-shrinkage temperature was significantly (*p* = 0.0018; **) higher after shrinkage (see Figure 11).

Of the 15 patients who required a full-thickness skin graft and in whom shrinkage was performed as part of the operation, 14 out of 15 (93%) had no complications. One patient who had a full-thickness skin graft did experience a complication in the context of minimal wound dehiscence, which is classified as Grade 1 according to the Clavien–Dindo classification. The wound dehiscence was treated conservatively and healed completely within two weeks. During the follow-up period of two to five months, no further complications occurred in these patients as a result of the shrinkage procedure.

## 4. Discussion

The authors are aware that shrinkage is an old and widely used technique. However, with this work, the authors aim to remind readers of this effective technique and encourage a renewed examination of its potential benefits.

Shrinkage can be advantageous in reducing wound tension. Excessive wound tension is known to negatively impact the healing process by impairing blood flow, leading to tissue ischemia, delayed healing, or even wound dehiscence. By adapting the wound edges through shrinkage, the mechanical stress on the wound edges can be alleviated, potentially improving blood flow and promoting tissue regeneration. This approach may also reduce the formation of hypertrophic scars, which are often associated with increased tension during the healing process [11].

The histological examinations demonstrated the typical histological changes through shrinkage. Our histological findings indicate the formation of a thermal scar and occurrence of collagen denaturation. Similar results occur after the usage of ablative lasers as mentioned by Ortiz et al. [12]. Within the areas of shrinkage, no vital fibrocytes can be detected and the collagen denaturation causes a “thickening and shortening of collagen fibrils” [12]. These findings can be seen in the early, as well in later, observations and, therefore, seem to be no limitation of these experiments. Furthermore, Manstein et al. found “[a] loss of dermal cell viability was co-localized with loss of collagen birefringence as assessed by cross polarization microscopy” when using “Fractional Photothermolysis” to create microscopic thermal injuries [13]. These observations correlate also with our findings. Collawn et al. found the collagen structure to be normal after one pass with a CO_2_-laser; fibroblast necrolysis was mostly present in the papillary dermis and the elastin was altered and mottled [14]. After multiple passes, the collagen compacted and fibril diameters became wider and spacing in between fibrils diminished, indicating that the impact of shrinkage rather correlates with multiple laser passes [14].

The assumed perpetuity of the induced shrinkage is not fully understood. Ortiz and Herron describe in their studies that the thermally altered tissue will be restructured and healed within three to six months through “new collagen deposition and fibroblast proliferation” as observed after usage of ablative lasers [12,13]. Chen et al. postulate that there is a recovery after the tissue has cooled down [15]. However, they also say that the long-term effects require further investigation [15]. Potzl et al. showed in their study that there was no full recovery after 9 weeks [16]. This time effect is sufficient, however, as the skin suture has healed after two weeks.

Using modern imaging techniques, we are able to measure the oxygenation of the tissue and the rise in temperature during shrinkage. We are aware, of course, that the use of diathermy results in an increase in temperature. However, we demonstrated with FlirOne that the average maximum temperature is around 65 °C. A study by Moran et al. shows that a temperature between 63 °C and 72 °C is required for shrinkage [17]. Although our average maximum temperature is at the lower end of the required temperature range, the clinical values indicate that a significant reduction in total area and width is achievable without complications.

These two non-invasive, easy-to-use, and radiation-free devices also enabled us to indirectly measure blood flow via the temperature and oxygen saturation of the tissue [10]. Shrinkage leads to a decrease in oxygen saturation in the tissue. However, this was only weakly significant [*p* = 0.0245 and 0.0148]. The authors’ clinical experience does not indicate an increased rate of wound healing disorders due to this slightly reduced oxygen saturation. This is also shown by the observed rate of complication-free outcomes, which was 93% in our study. It is debatable whether the case of the sole wound dehiscence was a possible postoperative outcome or whether the shrinkage was responsible for it. However, fourteen complication-free cases suggest that the one case of wound dehiscence at the donor site is the result of an increased tension.

The donor site for full-thickness skin grafts is rarely considered in the literature. In a study by Custer et al., a wound dehiscence rate of barely 4% (n = 2) was found [18]. In two patients (4%), a hypertrophic scar occurred during the course of the healing process [18]. As already mentioned, the increased tension of the wound edges can lead to this [11]. In summary, full-thickness skin harvesting without shrinkage has a complication rate of approximately 8% (n = 4). Thus, there is no difference to our study when using shrinkage. For this reason, the authors consider shrinkage to be a valuable method to reducing the wound area and, thus, the tension on the wound edges.

We would also like to point out the following: split-thickness skin grafts (STSG) and full-thickness skin grafts (FTSG) are the most commonly used types of grafts in reconstructive surgeries. While larger defects are often covered with split-thickness skin grafts, there are still certain cases where a full-thickness skin graft is indicated, e.g. when using cross-finger flaps for digital defect reconstruction [19]. Tissue engineering, especially generation of skin substitutes to replace the loss of skin, is a promising new tool in this context but is not yet part of the clinical practice [20].

However, the use of FTSG is limited by the primary closure of the donor site and is, therefore, mainly used for smaller defects. Several studies involving a total of 420 (174 FTSG vs. 258 STSG) grafts demonstrated that the rate of post-graft contracture was lower in full-thickness skin graft (FTSG) transplantation compared to STSG [15,21,22,23,24,25]. Accordingly, the need for surgical revision (surgical release) was also significantly lower in the FTSG group in six studies with a total of 408 (158 FTSG vs. 257 STSG) transplants [21,22,23,24,25,26,27].

Our results show an overall size reduction of almost 47%. Examining the reduction in wound width, we found a significant decrease of 36.5%. The results of our study demonstrate that significant wound reduction can be achieved using shrinkage, thereby the potential to allow harvesting of larger full-thickness skin grafts. Particularly considering that full-thickness skin grafts shrink to 91.5% of their original size after one year, it is useful to know the possible dimensions and how the limits of harvesting can be extended [28].

The authors hypothesize that shrinkage may improve both the functional and cosmetic outcomes of wound healing. However, objective assessment methods, such as cutometer measurements to assess skin elasticity and mechanical properties, are required to quantify these effects. The small number of patients and the lack of in vivo experiments are certainly a limitation of this study. Further studies to validate this approach are essential, and the authors are planning such trials, in particular to exclude possible biases due to intra- and interstation variability. Longitudinal studies assessing wound healing quality and scar tissue resilience could further solidify shrinkage as a reliable and safe method for wound management, particularly in surgeries where both functionality and appearance are crucial.

## 5. Conclusions

This study confirms that thermal-induced shrinkage effectively reduces wound size with an average wound area reduction of almost 47% through a temperature rise of 38.3 °C, despite a minor decrease in oxygen saturation, which did not impact healing. Histological findings underline the clinical results. Our study confirms that shrinkage extends the reduction in wound tension and feasibility of larger full-thickness skin grafts. In certain cases, this leads to a better result, since the risk of contracture is lower with full-thickness skin grafts.

## Data Availability

The raw data supporting the conclusions of this article will be made available by the authors, without undue reservation.
